# Orthopedic trauma is associated with higher serum concentrations of glial fibrillary acidic protein and ubiquitin C-terminal hydrolase L1 in mild traumatic brain injury with negative head computed tomography

**DOI:** 10.11613/BM.2026.010707

**Published:** 2026-02-15

**Authors:** Alma Osmić-Husni, Radivoj Jadrić

**Affiliations:** 1 Department for Biochemistry, Polyclinic for Laboratory Diagnostics, University Clinical Centre Tuzla, Tuzla, Bosnia and Herzegovina; 2 Department of Medical Biochemistry, Faculty of Medicine, University of Sarajevo, Sarajevo, Bosnia and Herzegovina

**Keywords:** GFAP, orthopedic injuries, UCH-L1, head CT

## Abstract

**Introduction:**

Glial fibrillary acidic protein (GFAP) and ubiquitin C-terminal hydrolase L1 (UCH-L1) are increasingly used biomarkers in the evaluation of mild traumatic brain injury (mTBI), primarily to reduce the frequent overuse of head computed tomography (head CT). However, their specificity may be compromised by orthopedic trauma, which commonly accompanies mTBI. The aim of this study was to assess whether orthopedic trauma is associated with higher serum concentrations of GFAP and UCH-L1 in CT-negative mTBI patients, thereby potentially reducing their specificity for detecting CT-positive mTBI.

**Materials and methods:**

This prospective observational study included 67 CT-negative mTBI patients, of whom 29 (0.43) had orthopedic trauma and 38 (0.57) had none. Blood samples were obtained within 12 hours of injury and serum concentrations of GFAP and UCH-L1 were measured using a chemiluminescent microparticle immunoassay (CMIA) on the Alinity analyzer, following the manufacturer’s instructions. Statistical analysis included Mann-Whitney U test, chi-square test, Kruskal-Wallis test, *post-hoc* Dunn’s test and logistic regression analysis with P < 0.05 considered significant.

**Results:**

Serum GFAP concentrations were significantly higher in patients with orthopedic injuries (median (IQR): 70.0 (30.8 to 226.5) pg/mL) than in those without (24.95 (5.52 to 49.15) pg/mL; P < 0.001). Similarly, UCH-L1 concentrations were higher in the orthopedic injury group (median (IQR): 2494.3 (670.1 to 5708.1) pg/mL) compared with those without trauma (262.8 (153.8-595.3) pg/mL; P < 0.001).

**Conclusions:**

Orthopedic trauma is associated with higher serum concentrations of GFAP and UCH-L1 in CT-negative mTBI patients, which may reduce the specificity of these biomarkers for ruling out intracranial injury.

## Graphical abstract

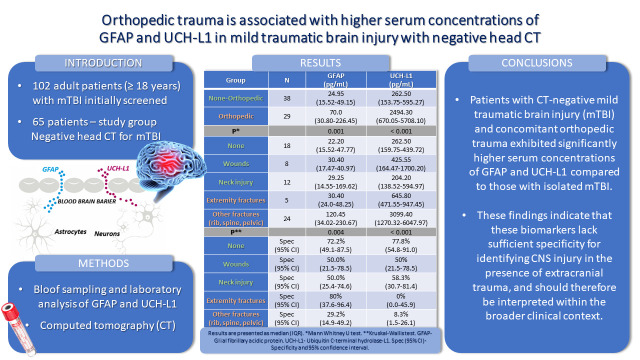

## Highlights

• Serum glial fibrillary acidic protein (GFAP) and ubiquitin C-terminal hydrolase L1 (UCH-L1) concentrations were measured in computed tomography (CT) - negative mild traumatic brain injury (mTBI)

• GFAP and UCH-L1 concentrations were higher in mTBI with orthopedic trauma

• GFAP and UCH-L1 concentrations had reduced specificity in CT-negative mTBI

• Orthopedic trauma is associated with higher GFAP and UCH-L1 in CT-negative mTBI

• Clinical interpretation of biomarkers should be integrated with patient assessment

## Introduction

Mild traumatic brain injury (mTBI) is the most common form of traumatic brain injury, accounting for approximately 70-90% of all TBI cases globally (*1*). Although most patients recover within days or weeks, a significant proportion of experience persistent cognitive, emotional, and physical symptoms lasting months or even years (*2*).

Clinically, mTBI is defined by a Glasgow coma scale (GCS) score of 13-15, transient neurological symptoms, and frequently, a normal head computed tomography (CT) scan (*3*). Despite the availability of validated clinical decision rules, CT remains widely overused in patients with mTBI, exposing many individuals to unnecessary ionizing radiation and increasing health-care costs (*4*). At the same time, a recent systematic review confirmed that CT is indispensable in the acute evaluation of TBI for the rapid identification of life-threatening lesions such as intracranial hemorrhage and skull fractures, which understandably makes clinicians reluctant to omit CT in the emergency setting (*5*).

In a large trauma cohort, both markers were rapidly detectable in serum within hours after injury and showed good diagnostic accuracy for CT-defined intracranial lesions and the need for neurosurgical intervention (*6*). In severe TBI, higher serum GFAP and UCH-L1 concentrations correlated with lower GCS scores, abnormal CT findings and mortality (*7*). In the ALERT-TBI multicenter trial, a combined GFAP/UCH-L1 test performed within 12 hours of injury showed very high sensitivity and negative predictive value for ruling out CT-detectable intracranial injuries (*8*). A recent single-centre study in patients with suspected mTBI reported similar performance, supporting the potential clinical role of these biomarkers in safely reducing unnecessary head CT scans (*9*). To support clinicians in the early evaluation of suspected mTBI, laboratory assays have been developed for routine use. The Abbott Alinity i TBI test is an *in vitro* diagnostic chemiluminescent microparticle immunoassay (CMIA) that provides quantitative measurement of GFAP and UCH-L1 in serum and plasma, intended for adults (≥ 18 years) within 12 hours of injury (GCS 13–15), using cut-off values of 35 pg/mL for GFAP and 400 pg/mL for UCH-L1; combined interpretation of both markers improves diagnostic accuracy and supports reduction of unnecessary CT scans (*10*).

Recent reviews emphasized that GFAP and UCH-L1 are among the most extensively validated biomarkers of brain injury, highlighting their potential clinical application. These reviews also pointed to important challenges, including the need for assay standardization, establishment of clinically relevant cut-off values, and further validation before routine implementation (*11*). A systematic review and meta-analysis from 2025 reported diagnostic performances for the individual biomarkers in identifying CT-positive mTBI, showing that GFAP reached a sensitivity of 76% and a specificity of 74%, while UCH-L1 demonstrated a higher sensitivity (86%) but a lower specificity (51%). These values apply to single biomarker performance and do not directly reflect the combined GFAP/UCH-L1 assay employed in this study (*12*). A recent prospective study conducted in an emergency department setting evaluated the diagnostic performance of GFAP and UCH-L1 in patients with mild traumatic brain injury (mTBI). The authors reported that GFAP achieved a sensitivity of 83.3% and a specificity of 37.9%, while UCH-L1 showed a sensitivity of 50.0% and a specificity of 65.0%. These findings indicate that, although both biomarkers demonstrate potential for rapid triage of mTBI, further validation is required before their implementation as standard adjuncts to CT (*13*). A large European multicenter study further demonstrated that measurement of GFAP and UCH-L1 can effectively exclude intracranial lesions in patients with mTBI, supporting their diagnostic utility in clinical decision-making (*14*).

Despite promising sensitivity, the specificity of these biomarkers in real world scenarios remains uncertain, particularly in patients with extracranial injuries, such as orthopedic trauma. In a prospective two-center study, 73 patients with acute orthopedic trauma without head injury were compared to 93 patients with CT-negative mTBI. GFAP concentrations on admission were significantly higher in orthopedic trauma patients than in CT-negative mTBI, while UCH-L1 concentrations did not differ between the groups. Importantly, magnetic resonance imaging (MRI) performed in 71% of the orthopedic cohort revealed no acute traumatic lesions, confirming higher serum concentrations of GFAP and UCH-L1 despite the absence of brain injury (*15*). In a prospective cohort of adult trauma patients, GFAP showed higher accuracy than S100β for detecting traumatic intracranial lesions on CT and remained reliable even in the presence of extracranial fractures, whereas S100B concentrations were markedly influenced by extracranial injuries (*16*). Another study demonstrated that early serum concentrations of GFAP breakdown products (GFAP-BDP), measured within 4 hours after injury, reliably discriminated TBI patients from trauma and healthy controls and were associated with intracranial CT lesions as well as the need for neurosurgical intervention (*17*). A methodological article on TBI biomarker research stressed that many published studies are limited by small samples, heterogeneous patient cohorts and suboptimal timing and handling of blood samples, and that rigorous standardization is essential for correct interpretation of GFAP and UCH-L1 results (*18*). However, data on CT-negative mTBI with concomitant orthopedic trauma are still limited. Therefore, the aim of our prospective observational study was to assess whether orthopedic trauma is associated with higher concentrations of GFAP and UCH-L1 in CT-negative mTBI patients and whether this may affect the specificity of these biomarkers for detecting intracranial injury.

## Materials and methods

### Study design

This prospective observational study was conducted at the University Clinical Center (UCC) Tuzla over an eight-month period (August 2024-March 2025). Adult patients (≥ 18 years) presenting with mTBI were screened for eligibility. Mild TBI was defined as head injury with a GCS score of 13–15 on admission, in accordance with a widely accepted definition of mild traumatic injury (*19*, *20*). The study protocol was reviewed and approved by the Ethics Committee of UCC Tuzla (Approval No. 02-09/2-114/23) and written informed consent was obtained from all participants prior to inclusion.

### Subjects

Inclusion criteria were age ≥ 18 years, hospital admission within 12 hours post-injury, GCS 13–15, availability of both serum biomarker testing (GFAP and UCH-L1) and head CT imaging performed on admission. Exclusion criteria included GCS < 13, penetrating head trauma, venous blood sampling conducted more than 12 hours after injury, and patients with acute intracranial findings on head CT (CT-positive mTBI). Patients with no acute findings on head CT were further categorized into two groups based on the presence or absence of orthopedic injuries, to assess whether such injuries contribute to elevated serum concentrations of GFAP and UCH-L1 in the absence of CT-positive findings.

Patients presenting with head injury were initially assessed in the emergency surgical department. Mild TBI was diagnosed in patients with aGCS score of 13-15 on admission and at least one of the following: transient loss of consciousness (< 30 min), post-traumatic amnesia (< 24 h), or transient confusion/disorientation, consistent with a widely accepted definition of mTBI (*20*). The decision to perform head CT was made by the on-duty surgeon according to the standard emergency department protocol, which considered clinical risk factors such as loss of consciousness, post-traumatic amnesia, repeated vomiting, severe headache, seizure, or signs of skull fracture. Computed tomography scans were performed by radiology engineers and interpreted by board-certified radiologists within one hour of imaging. A CT-positive finding was defined as the presence of intracranial hemorrhage (subarachnoid, subdural, epidural, or intracerebral), cerebral contusions, or skull fractures. Patients without acute intracranial findings were classified as CT-negative and were eligible for our study group. Orthopedic injuries were confirmed both radiologically (X-ray or CT) and by orthopedic specialists and included long bone fractures (femur, tibia, humerus, radius/ulna), pelvic fractures, and fractures of the upper and lower extremities such as clavicle, hand, or foot bones. The mechanism of injury was recorded for all patients.

### Methods

Venous blood samples were collected upon hospital admission and in all cases within 12 hours of the traumatic event. Blood was drawn into 6 mL serum tubes with clot activator (Vacusera CAT Serum, Disera A.Ş., İzmir, Turkey) and immediately transported to the Department of Biochemistry at the UCC Tuzla Polyclinic for Laboratory Diagnostics for processing. In the laboratory, samples were allowed to clot for approximately 30 minutes at room temperature (total time from venipuncture to centrifugation ≈ 30 minutes), followed by centrifugation at 3000 rpm for 7 minutes (≈ 21 000 g-minutes) in accordance with the manufacturer’s instructions for the Alinity i TBI assay (Abbott diagnostics, Abbott Park, USA), to ensure complete separation of serum and removal of cellular components (*10*). Prior to analysis, all serum samples were visually inspected for hemolysis, lipemia, and icterus. Samples exhibiting visible discoloration or turbidity were excluded from testing to avoid analytical interference. The hemolysis, icterus and lipemia (HIL) index detection module was not enabled on the Alinity i analyzer, therefore visual inspection served as the preanalytical quality-control step. All biomarker analyses were performed within two hours after centrifugation.

Serum concentrations of GFAP and UCH-L1 were quantified using chemiluminescent microparticle immunoassay (CMIA) technology on the Alinity i analyzer (Abbott diagnostics, Abbott Park, USA). The analyses employed GFAP Reagent Kit (04W17) and UCH-L1 Reagent Kit (04W19). Calibration was performed using the respective GFAP Calibrators (04W1701) and UCH-L1 Calibrators (04W1910) and verified by two levels of internal control materials (GFAP Controls 04W1710; UCH-L1 Controls 04W1910). Calibration and control testing followed the Abbott assay protocol (*10*). External quality-control material was not applied, as the assay was conducted exclusively for research purposes and is not part of the routine diagnostic workflow of the laboratory. Analytical performance characteristics were consistent with manufacturer specifications: analytical measuring interval (AMI): 6.1-42 000 pg/mL (GFAP); 26.3-25 000 pg/mL (UCH-L1); limit of detection (LoD): 3.2 pg/mL (GFAP); 18.3 pg/mL (UCH-L1) and intra-assay CV < 6%; Inter-assay CV < 8%.

Values below the LoD were reported as “< LoD” and excluded from statistical analysis. Diagnostic cut-off values were 35 pg/mL for GFAP and 400 pg/mL for UCH-L1, as recommended in the Alinity i TBI assay documentation. Samples exceeding either threshold were interpreted as positive (*10*).

### Statistical analysis

Descriptive statistics were reported as median with interquartile range (IQR; Q1-Q3) for non-normally distributed variables, as determined using the Shapiro-Wilk test, which was applied to assess the normality of data distribution. Categorical variables were presented as ratios. Group comparisons were performed using the Mann–Whitney U test for comparisons between two independent groups, and the Kruskal-Wallis test for comparisons involving three or more groups, as both tests are appropriate for continuous non-parametric data and do not assume normality. Differences in categorical variable frequencies were assessed using the χ^2^ (chi-square) test while all expected cell counts were sufficient (≥ 5), which allows efficient evaluation of associations between categorical variables in larger datasets. *Post-hoc* comparisons between injury subtypes were conducted using Dunn’s test, following significant Kruskal-Wallis results, to identify specific group differences while controlling for multiple comparisons. Adjusted P-values from Dunn’s *post-hoc* tests were reported using the Bonferroni correction method to control for the family-wise error rate across multiple subgroup comparisons. In addition to P-values, effect sizes were quantified by the Hodges–Lehmann median differences between groups, with corresponding 95% confidence intervals (95% CI), providing a robust measure of the magnitude and direction of observed differences. Multivariable linear regression on log-transformed biomarker concentrations was used to adjust for confounding factors. To assess independent effects, multivariable logistic regression models were applied. All statistical tests were two-tailed, and a P-value < 0.05 was considered statistically significant. Statistical analysis was performed using IBM SPSS Statistics for Windows, Version 23.0 (IBM Corp., Armonk, USA).

## Results

A total of 102 adult patients with mTBI were initially screened. Thirty four patients were excluded due to positive head CT findings, and one patient was excluded because of hemolysis, leaving 67 head CT-negative patients who met the inclusion criteria and were included in the final analysis. Of these, 29/67 had orthopedic injuries while the remaining 38/67 had no such injuries.

Serum biomarker, age and time from injury/blood draw analysis (Table 1) revealed significantly higher concentrations of both GFAP and UCH-L1 in the orthopedic injury group compared to the non-orthopedic group. GFAP concentrations had a median of 70.0 (30.80-226.45) pg/mL *versus* 24.95 (15.52-49.15) pg/mL (P = 0.001), while UCH-L1 concentrations were 2494.3 (670.05-5708.10) pg/mL *versus* 262.5 (153.75-595.27) pg/mL (P < 0.001). Age showed a significant difference between orthopedic injury group with median (min-max) 51 (18-77) years and the non-orthopedic group with median 33 (18-64) years, P = 0.017. Time from injury/blood draw (hours) did not show a significant difference between orthopedic injury group with median 3.0 (2.0-4.0) hours, and the non-orthopedic group with median 3.0 (2.0-3.5) hours, P = 0.155.

**Table 1:** Differences in continuous variables between head CT-negative mild traumatic brain injury patients with and without orthopedic injuries

**Parameter**	**Injuries**	**N**	**Median**	**Range**		**P**	**adjusted β** **(95% CI)**	**P** **(adjusted)**
Age (years)	Non-Ort	38	33	18	64	0.017	/	/
	Orthopedic	29	51	18	77			
Time (h)	Non-Ort	38	3.0	2.0	4.0	0.155	/	/
	Orthopedic	29	3.0	2.0	3.5			
GFAP (pg/mL)	Non-Ort	38	24.95	15.52	49.15	0.001	1.80 (1.20 to 2.40)	< 0.001
	Orthopedic	29	70.0	30.80	226.45			
UCH-L1 (pg/mL)	Non-Ort	38	262.50	153.75	595.27	< 0.001	1.90 (1.30 to 2.50)	< 0.001
	Orthopedic	29	2494.30	670.05	5708.10			
Age is presented as median (min-max). CT - computed tomography. Non-Ort - non orthopedic. Time - time from injury/blood draw. GFAP - glial fibrillary acidic protein. UCH-L1 - ubiquitin C-terminal hydrolase L1. CI - confidence interval. P < 0.05 was considered statistically significant.								

In age-adjusted linear regression, orthopedic injury remained independently associated with higher GFAP (β 1.80, 95% CI 1.20-2.40; P < 0.001) and UCH-L1 concentrations (β 1.90, 95% CI 1.30-2.50; P < 0.001). Additional analyses showed no significant associations of sex, injury mechanism or time from injury with biomarker concentrations, and these variables were therefore not retained as confounders.

When patients were stratified according to injury type, biomarker values again differed across subgroups (Table 2). GFAP and UCH-L1 concentrations were lower in patients without additional injuries and higher in those with fractures, particularly rib, spine or pelvic fractures, and in patients with neck injuries (P = 0.004 for GFAP and P < 0.001 for UCH-L1). *Post-hoc* Dunn’s tests with Bonferroni correction (Table 3) showed that patients with rib/spine/pelvic fractures had significantly higher GFAP and UCH-L1 concentrations than those without additional injuries, with median differences and 95% CI presented as effect sizes.

**Table 2:** Differences in biomarker values among head CT-negative mild traumatic brain injury (mTBI) patients according to injury type

**Biomarker**	**Group**	**Type of injuries**	**N**	**Median**	**IQR**		**P**
	Non-Ort	None	18	22.20	15.52	47.77	
		Wounds	8	30.40	17.47	40.97	
GFAP (pg/mL)		Neck	12	29.25	14.55	169.62	0.004
	Orthopedic	Extr. fract.	5	30.40	24.0	48.25	
		Other fract.	24	120.45	34.02	230.67	
	Non-Ort	None	18	262.50	159.75	439.72	
		Wounds	8	425.55	164.47	1700.20	
UCH-L1 (pg/mL)		Neck	12	204.20	138.52	594.97	< 0.001
	Orthopedic	Extr. fract.	5	645.80	471.55	947.45	
		Other fract.	24	3099.40	1270.32	6047.97	
CT - computed tomography. Non-Ort - non orthopedic. None - no injuries. Extr.fract. - extremity fractures. Other fract. - fractures of ribs, spine or pelvic bones. Neck - neck injuries, whiplash. Time - time from injury/blood draw. GFAP - glial fibrillary acidic protein. UCH-L1 - ubiquitin C-terminal hydrolase L1. IQR - interquartile range. P < 0.05 was considered statistically significant.							

**Table 3:** *Post-hoc* Dunn’s test of GFAP and UCH-L1 differences between injury subgroups in head CT-negative mild traumatic brain injury patients

	**GFAP**			**UCH-L1**		
Group pairs	MD (HL)	95% CI	P (Bonf)	MD (HL)	95% CI	P (Bonf)
None - Wounds	- 3.604	- 20.01 to 12.80	1.000	- 7.889	- 22.06 to 7.12	1.000
None - Extr. fract.	- 5.917	- 24.00 to 12.17	1.000	- 15.439	- 31.67 to 0.20	0.590
None - Neck	- 6.208	- 22.56 to 13.53	1.000	- 2.014	-1 5.95 to 12.43	1.000
None - Other fract.	- 22.146	- 38.33 to - 8.18	0.020	- 31.222	- 44.68 to - 18.70	< 0.010
Wounds -Extr. fract.	2.313	- 15.95 to 23.89	1.000	7.550	- 9.49 to 25.70	1.000
Wounds - Neck	- 2.604	- 18.71 to 14.44	1.000	5.875	- 9.21 to 22.20	1.000
Wounds - Other fract.	18.542	4.29 to 34.18	0.200	23.333	9.19 to 36.43	0.030
Extr. fract. - Neck	- 0.292	- 19.92 to 19.28	1.000	13.425	- 4.24 to 30.00	1.000
Extr. fract.- Other fract.	- 16.229	- 34.36 to 2.76	1.000	- 15.783	- 32.32 to 1.32	0.590
Neck - Other fract.	15.938	3.26 to 31.72	0.210	29.208	15.03 to 44.60	< 0.010
CT - computed tomography. None - no injuries. Extr.fract. - extremity fractures. Other fract. - fractures of ribs, spine or pelvic bones. Neck - neck injuries, whiplash. MD(HL) - median difference (Hodges-Lehmann). P(Bonf) - Bonferroni adjusted P-value. Note: Bonferroni adjustment for 10 comparisons. GFAP - glial fibrillary acidic protein. UCH-L1 - ubiquitin C-terminal hydrolase L1. CI - confidence interval. P < 0.05 was considered statistically significant.						

In adjusted logistic regression including GFAP, UCH-L1 and age, higher UCH-L1 concentrations (OR 1.001, 95% CI 1.000-1.002; P = 0.006) and higher age (OR 1.056, 95% CI 1.011-1.104; P = 0.015) were independently associated with orthopedic injury, whereas GFAP (OR 1.006, 95% CI 0.999-1.012; P = 0.088) did not retain statistical significance (Table 4).

**Table 4:** Association of blood biomarkers with orthopedic trauma in head CT-negative mild traumatic brain injury patients

**Predictor**	**Adjusted logistic regression**		
	**OR**	**95% CI**	**P**
GFAP	1.006	0.999 to 1.012	0.088
UCH-L1	1.001	1.000 to 1.002	0.006
Age	1.056	1.011 to 1.104	0.015
CT - computed tomography. OR - odds ratio. GFAP - glial fibrillary acidic protein. UCH-L1 - ubiquitin C-terminal hydrolase L1. CI - confidence interval. P < 0.05 was considered statistically significant.			

Among CT-negative mTBI patients, test specificity also varied by injury type (Table 5). GFAP specificity was higher in patients without additional injuries and in those with extremity fractures (72.2% and 80.0%, respectively), and lower in patients with other fractures (29.2%), wounds or neck injuries (both 50.0%). For UCH-L1, specificity was high in patients without additional injuries (77.8%) but markedly lower in those with extremity and other fractures (0% and 8.3%, respectively).

**Table 5:** Test specificity among head CT-negative mild traumatic brain injury patients according to injury type

**Test**		**Orthopedic**		**Non - Orthopedic**		
		**Extremity fractures**	**Other fractures**	**None**	**Wounds**	**Neck injury**
	Positive	1	17	5	4	6
	Negative	4	7	13	4	6
GFAP	Total	5	24	18	8	12
	Specificity (%)	80	29.2	72.2	50	50
	95% CI	37.6-96.4	14.9-49.2	49.1-87.5	21.5-78.5	25.4-74.6
	Positive	5	22	4	4	5
	Negative	0	2	14	4	7
UCH-L1	Total	5	24	18	8	12
	Specificity (%)	0	8.3	77.8	50	58.3
	95% CI	0.0-45.9	1.5-26.1	54.8-91.0	21.5-78.5	30.7-81.4
CT - computed tomography. None - no injuries. Other fract. - fractures of ribs, spine or pelvic bones. Neck - neck injuries, whiplash. GFAP - glial fibrillary acidic protein. UCH-L1 - ubiquitin C-terminal hydrolase L1. CI - confidence interval.						

## Discussion

In this prospective observational single-centre study, serum concentrations of GFAP and UCH-L1 in CT-negative patients with mild traumatic brain injury (mTBI) were significantly higher in those with concomitant orthopedic injuries than in those without such trauma. These findings suggest that orthopedic trauma may contribute to additional release of these biomarkers, even in the absence of intracranial pathology visible on CT imaging.

Participants in the orthopedic group were older, and the groups also differed in sex distribution; however, additional analyses showed no meaningful influence of sex, GCS score or time from injury to sampling on biomarker concentrations. In age-adjusted models, the presence of orthopedic trauma remained associated with higher GFAP and UCH-L1 concentrations, indicating that concomitant extracranial orthopedic injury, rather than demographic or procedural factors, is the main driver of biomarker elevation in CT-negative mTBI.

Within the CT-negative cohort, biomarker concentrations also varied according to injury type. Concentrations of GFAP and UCH-L1 were lowest in patients without extracranial injuries and tended to be higher in those with fractures, particularly rib, spine or pelvic fractures, consistent with the overall differences detected by non-parametric tests. *Post-hoc* Dunn’s tests with Bonferroni correction identified only a limited number of significant pairwise differences, for example between patients with rib/spine/pelvic fractures and those without extracranial injuries for GFAP, and for UCH-L1 also versus some non-orthopedic subgroups such as neck injuries and soft-tissue wounds. However, many comparisons between fracture and non-orthopedic subgroups did not reach statistical significance, so these patterns should be interpreted with caution. Overall, the data suggests a trend towards higher GFAP and UCH-L1 concentrations in patients with more extensive extracranial injury, rather than a uniform and statistically robust gradient across all injury subgroups.

The strong positive correlation between GFAP and UCH-L1 indicates that these biomarkers show a largely parallel response after trauma. However, only UCH-L1 retained an independent association with orthopedic trauma in multivariable logistic regression adjusted for age, whereas GFAP did not remain significant. Taken together, these findings suggest that UCH-L1 may represent a more robust marker of concomitant orthopedic injury in CT-negative mTBI, while GFAP appears to be more susceptible to confounding and to contribute less independently to the overall extracranial injury profile.

In our CT-negative mTBI cohort, both GFAP and UCH-L1 showed only moderate specificity, with pronounced variability across extracranial injury patterns. This pattern is broadly consistent with findings from a large prospective cohort study in a mixed trauma population, a systematic review and meta-analysis in patients with mild TBI, a multicentre observational study of mild TBI, an emergency department–based diagnostic study, and a European multicentre study, all of which reported high sensitivity and negative predictive value for CT-positive lesions but only moderate specificity (*6*, *8*, *12*-*14*). However, these studies rarely quantified specificity separately in CT-negative patients or according to the presence and type of extracranial trauma. Our data add to this evidence by showing that, within CT-negative mTBI, specificity is highest in patients without extracranial injuries (GFAP 72.2%, UCH-L1 77.8%) or with isolated extremity fractures (GFAP 80.0%), and drops markedly in those with fractures of the ribs, spine or pelvis (GFAP 29.2%, UCH-L1 8.3%), wounds, or neck injuries. These findings provide more granular estimates of how different extracranial injury patterns modify the diagnostic performance of these biomarkers in the CT-negative population that is most relevant for clinical decision-making.

Only a limited number of previous reports have directly examined how extracranial trauma influences glial biomarker concentrations. In a general trauma cohort with a high proportion of patients who sustained extracranial fractures involving the torso and extremities, these fractures substantially reduced the specificity of S100B, whereas GFAP maintained relatively good accuracy for detecting intracranial lesions on CT and was less affected by fractures (*16*). In our CT-negative mTBI cohort, GFAP specificity was likewise highest in patients without extracranial injuries and in those with isolated limb fractures, but decreased in patients with rib, spine or pelvic fractures, wounds and neck injuries, while UCH-L1 specificity dropped even more markedly across these orthopedic subgroups, particularly in those with rib, spine or pelvic fractures.

There is one prospective two-centre study that directly compared CT-negative mTBI patients with orthopedic trauma controls without head injury (*15*). In that study, serum GFAP and UCH-L1 were measured repeatedly from admission up to several months after injury and related to neuroimaging findings in 73 patients with acute orthopedic trauma and 93 patients with CT-negative mTBI. At admission, GFAP concentrations were higher in orthopedic trauma than in CT-negative mTBI, whereas UCH-L1 concentrations did not differ at any time point, and neither biomarker was able to distinguish CT-negative mTBI from orthopedic trauma; patients with orthopedic injuries and elevated GFAP or UCH-L1 were therefore considered at risk of being misclassified as having concomitant mTBI and of undergoing unnecessary brain imaging, and the authors questioned the diagnostic value of these markers for mild CT-negative TBI (*15*). A methodological commentary later pointed out that heterogeneous inclusion criteria, incomplete early sampling and possible misclassification of mild TBI cases within the orthopedic group may have contributed to these findings (*18*). By comparison, our study is restricted exclusively to CT-negative mTBI, using a single early blood sample obtained within 12 h of injury on one commercial platform, and stratifies patients according to the presence and pattern of orthopedic injuries rather than including trauma controls without head injury. We also found higher GFAP and UCH-L1 concentrations in patients with orthopedic injuries, and in age-adjusted models both biomarkers remained independently associated with orthopedic injury. These findings support the view that extracranial trauma is an important source of biomarker positivity in CT-negative mTBI and may explain part of the biomarker-positive/CT-negative cases reported in large diagnostic trials (*6*, *8*, *12*–*15*, *20*).

Histological studies have demonstrated that GFAP is not confined to astrocytes but is also expressed in Schwann cells, satellite cells and enteric glia, as well as in fibroblasts, chondrocytes, bone marrow stromal cells, periosteum and cardiac valves, providing multiple potential extracranial sources after orthopedic trauma (*21*, *22*). Our findings are consistent with these concerns, as we show that the presence and localization of orthopedic injuries substantially reduce the specificity of both GFAP and UCH-L1 in CT-negative mTBI, whereas specificity is highest in patients without extracranial injuries. Taken together with large-scale diagnostic and methodological work, our results support the use of GFAP/UCH-L1 primarily as high-sensitivity rule out tools and indicate that positive biomarker results should be interpreted with particular caution when major extracranial trauma is present (*6*, *8*, *12*–*15*, *20*).

This study has several limitations that should be considered when interpreting specificity results. Biomarker concentrations were measured once during the acute phase, preventing assessment of temporal dynamics or delayed release patterns. Lack of MRI correlation was another limitation, as MRI was not routinely performed, which limited verification of microstructural brain lesions in CT negative cases and may have led to misclassification of some biomarker positive patients. Small subgroup sizes also posed a limitation, as certain extracranial injury categories such as wounds and neck injuries included a limited number of participants, resulting in wider confidence intervals. Minor timing and analytical variations were also possible. Although all samples were collected within 12 hours post-injury, small differences in timing and assay sensitivity may have affected absolute concentration. Potential confounding factors such as age, medications, and comorbidities could have influenced biomarker concentrations independently of trauma. The absence of a control group and follow up outcomes further limits interpretation. Without healthy controls or long-term data, prognostic interpretation remains limited. A recent review summarized several unresolved issues concerning the clinical use of blood biomarkers in mild traumatic brain injury, including uncertainties related to biomarker kinetics, specificity, and extracerebral sources (*23*). The present study directly addresses some of these questions by demonstrating that extracranial injuries substantially affect serum GFAP and UCH-L1 concentrations, limiting their diagnostic specificity in CT-negative mTBI.

In conclusion, our study demonstrated that patients with CT-negative mTBI and concomitant orthopedic trauma exhibited significantly higher serum concentrations of GFAP and UCH-L1 compared to those with isolated mTBI. These findings indicate that these biomarkers lack sufficient specificity for identifying CNS injury in the presence of extracranial trauma and should therefore be interpreted within the broader clinical context.

## Data Availability

The data generated and analyzed in the presented study are available from the corresponding author on request
